# Supplementary material for: Complete genome sequencing of SARS-CoV-2 strains that were circulating in Uzbekistan over the course of four pandemic waves
Source: PLoS One. 2024 Nov 19;19(11):e0298940. doi: 10.1371/journal.pone.0298940 (PMC11575833; doi:10.1371/journal.pone.0298940)
Supplement: S1 Table — (DOCX) [file pone.0298940.s001.docx]

|  | **Amino acid changes in the nonstructural proteins and accessory proteins** | **Total number of sequenced samples 110** | **Frequency** | **20A** | **Alpha** | **Delta** | **Omicron** |
| --- | --- | --- | --- | --- | --- | --- | --- |
| **NSP1** | **S135R** | 60 | 55% | 0 | 0 | 0 | 59 |
|  | **K47R** | 11 | 10% | 0 | 0 | 0 | 11 |
|  | **G82D** | 2 | 2% | 0 | 0 | 0 | 1 |
|  | **M85del** | 2 | 2% |  |  |  |  |
|  | **D139Y, F143del, G133C, K141del, Q66R, R24C, S142del** | 1 | 1% | 0 | 0 | 0 | 1 |
|  | **R124C** | 1 | 1% | 0 | 0 | 1 | 0 |
| **NSP2** | **R27C** | 22 | 20% | 2 | 2 | 18 | 0 |
|  | **K81N** | 8 | 7% | 0 | 0 | 8 | 0 |
|  | **P129L** | 7 | 6% | 0 | 0 | 7 | 0 |
|  | **E563D, K142R, K554E** | 2 | 2% | 0 | 0 | 0 | 2 |
|  | **L550F** | 2 | 2% | 0 | 0 | 2 | 0 |
|  | **G339S, M141R** | 1 | 1% | 0 | 1 | 0 | 0 |
|  | **D23Y, R119K, T547I, T590I** | 1 | 1% | 0 | 0 | 1 | 0 |
|  | **D43Y, I295V, P129S, Q376K, Q395R, S348P** | 1 | 1% | 0 | 0 | 0 | 1 |
| **NSP3** | **G489S** | 60 | 55% | 0 | 0 | 60 | 0 |
|  | **T24I** | 58 | 53% | 0 | 0 | 58 | 0 |
|  | **P1469S** | 23 | 21% | 0 | 23 | 0 | 0 |
|  | **P1228L** | 22 | 20% | 0 | 22 | 0 |  |
|  | **A488S** | 21 | 19% | 0 | 21 | 0 | 0 |
|  | **P822L** | 12 | 11% | 0 | 12 | 0 | 0 |
|  | **H1274Y** | 9 | 8% | 0 | 9 | 0 | 0 |
|  | **S126L** | 8 | 7% | 0 | 8 | 0 | 0 |
|  | **E374G** | 4 | 4% | 0 | 0 | 4 | 0 |
|  | **I1412T** | 4 | 4% | 4 | 0 | 0 | 0 |
|  | **T183I** | 3 | 3% | 3 | 0 | 0 | 0 |
|  | **A1711V, E1799G** | 2 | 2% | 0 | 0 | 2 | 0 |
|  | **A579V, T73I** | 2 | 2% | 0 | 2 | 0 | 0 |
|  | **A890D, T771I** | 2 | 2% | 2 | 0 | 0 | 0 |
|  | **E113D, L632F, Q585H, S1670F, T1036I, T1866A, V1243F, Y103N** | 1 | 1% | 0 | 0 | 1 | 0 |
|  | **A1310V, A1892T, G1716S, I388T, K38R, L1266I, M375I, P822S, R568H, S1265del, S403L, S692F, V1139I, V1522I, V665I, V929I** | 1 | 1% | 0 | 0 | 0 | 1 |
| **`NSP4** | **T492I** | 80 | 73% | 0 | 0 | 21 | 59 |
|  | **T327I** | 57 | 52% | 0 | 0 | 0 | 57 |
|  | **L264F** | 56 | 51% | 0 | 0 | 0 | 56 |
|  | **V167L** | 21 | 19% | 0 | 0 | 21 | 0 |
|  | **A446V** | 15 | 14% | 0 | 1 | 14 | 0 |
|  | **L438F** | 15 | 14% | 0 | 0 | 0 | 15 |
|  | **T96I** | 2 | 2% | 0 | 0 | 1 | 1 |
|  | **A146V, D279N, I110V, T189I, T214I** | 1 | 1% | 0 | 0 | 0 | 1 |
|  | **E141G** | 1 | 1% | 0 | 0 | 1 | 0 |
| **NSP5** | **P132H** | 59 | 54% | 0 | 0 | 0 | 59 |
|  | **K90R** | 2 | 2% | 0 | 0 | 1 | 1 |
|  | **A193V** | 1 | 1% | 0 | 1 | 0 | 0 |
|  | **A260V** | 1 | 1% | 0 | 0 | 1 | 0 |
|  | **T225I** | 1 | 1% | 0 | 0 | 0 | 1 |
| **NSP6** | **G107del, S106del** | 71 | 65% | 3 | 3 | 5 | 60 |
|  | **F108del** | 70 | 64% | 3 | 3 | 5 | 59 |
|  | **T77A** | 22 | 20% | 3 | 0 | 22 | 0 |
|  | **V149A** | 12 | 11% | 0 | 0 | 12 | 0 |
|  | **L75F** | 8 | 7% | 0 | 0 | 8 | 0 |
|  | **F34S, G277S, L237M, V149I** | 1 | 1% | 0 | 0 | 1 | 0 |
|  | **I189V, L105del, L260F, T10I, T181I, V152F** | 1 | 1% | 0 | 0 | 0 | 1 |
| **NSP7** | **L56F** | 2 | 2% | 0 | 0 | 2 | 0 |
| **NSP8** | **N118S** | 1 | 1% | 0 | 0 | 1 | 0 |
|  | **N192K, V160L** | 1 | 1% | 0 | 1 | 0 | 0 |
| **NSP9** | **T24I** | 1 | 1% | 0 | 0 | 1 | 0 |
| **NSP10** | **I55T** | 1 | 1% | 0 | 0 | 1 | 0 |
| **NSP12** | **P323L** | 108 | 98% | 3 | 4 | 41 | 60 |
|  | **G671S** | 53 | 49% | 0 | 1 | 39 | 13 |
|  | **A423V** | 2 | 2% | 0 | 0 | 2 | 0 |
|  | **L648F** | 2 | 2% | 0 | 0 | 0 | 2 |
|  | **P227L, P323F, P323F, V96I, Y479N** | 1 | 1% | 0 | 0 | 1 | 0 |
|  | **T225I, V792I, Y273H** | 1 | 1% | 0 | 0 | 1 | 0 |
| **NSP13** | **R392C** | 60 | 55% | 0 | 0 | 0 | 60 |
|  | **P77L** | 43 | 39% | 2 | 0 | 41 | 0 |
|  | **T127N** | 41 | 37% | 0 | 0 | 0 | 41 |
|  | **S36P** | 11 | 10% | 0 | 0 | 0 | 11 |
|  | **S38L** | 4 | 4% | 1 | 3 | 0 | 0 |
|  | **I399V** | 2 | 2% | 0 | 2 | 0 | 0 |
|  | **A85P, F24S, G206C, L295I, L297F, V169F** | 1 | 1% | 0 | 0 | 1 | 0 |
|  | **M233I, N102S, N268S, T481M, Y217H** | 1 | 1% | 0 | 0 | 0 | 1 |
| **NSP14** | **I42V** | 61 | 55% | 0 | 0 | 0 | 61 |
|  | **A394V** | 25 | 23% | 0 | 0 | 25 | 0 |
|  | **P46L** | 4 | 4% | 0 | 0 | 4 | 0 |
|  | **S461P** | 4 | 4% | 0 | 1 | 3 | 0 |
|  | **A471V** | 2 | 2% | 0 | 0 | 2 | 0 |
|  | **A1S, D144N, E204D** | 1 | 1% | 0 | 0 | 1 | 0 |
| **NSP15** | **T112I** | 58 | 53% | 0 | 0 | 0 | 58 |
|  | **H234Y** | 7 | 4% | 0 | 0 | 7 | 0 |
|  | **E56D, G286C, S147G V165I, V320L, V66L** | 1 | 4% | 0 | 0 | 1 | 0 |
| **NSP16** | **K160R** | 7 | 6% | 0 | 0 | 7 | 0 |
|  | **K182N** | 1 | 1% | 0 | 0 | 1 | 0 |
| **NS3** | **T223I** | 60 | 55% | 0 | 0 | 0 | 60 |
|  | **S26L** | 42 | 38% | 0 | 0 | 41 | 0 |
|  | **K16T** | 7 | 6% | 0 | 0 | 7 | 0 |
|  | **A72T** | 2 | 2% | 0 | 0 | 0 | 2 |
|  | **G172R** | 2 | 2% | 0 | 0 | 2 | 0 |
|  | **Q185H** | 2 | 2% | 0 | 2 | 0 | 0 |
|  | **F43C** | 1 | 1% | 1 | 0 | 0 | 0 |
|  | **G224C, T64I, T89I, V202L, Y113H** | 1 | 1% | 0 | 0 | 1 | 0 |
|  | **A99V, E181K, H78Y, L41R, Q38R, Y107H** | 1 | 1% | 0 | 0 | 0 | 1 |
| **NS6** | **I14V** | 1 | 1% | 0 | 0 | 0 | 1 |
|  | **T21I, V24I** | 1 | 1% | 0 | 0 | 1 | 0 |
| **NS7a** | **T120I** | 38 | 35% | 0 | 0 | 38 | 0 |
|  | **V82A** | 29 | 26% | 0 | 0 | 29 | 0 |
|  | **L116F** | 14 | 13% | 0 | 0 | 14 | 0 |
|  | **P45L** | 8 | 7% | 0 | 0 | 8 | 0 |
|  | **H47Y** | 1 | 1% | 0 | 0 | 0 | 1 |
|  | **R89I, V29L** | 1 | 1% | 0 | 0 | 1 | 0 |
| **NS7b** | **T40I** | 22 | 20% | 0 | 1 | 21 | 0 |
|  | **I27T** | 1 | 1% | 0 | 0 | 1 | 0 |
| **NS8** | **K68stop, Y73C** | 4 | 4% | 0 | 4 | 0 | 0 |
|  | **Q27stop** | 3 | 3% | 0 | 3 | 0 | 0 |
|  | **G8stop** | 2 | 2% | 0 | 0 | 0 | 2 |
|  | **F41C, I71F** | 1 | 1% | 0 | 0 | 0 | 1 |
|  | **R52I** | 1 | 1% | 0 | 1 | 0 | 0 |
|  | **T26I, T87I** | 1 | 1% | 0 | 0 | 1 | 0 |
