# Supplementary material for: Complete genome sequencing of SARS-CoV-2 strains that were circulating in Uzbekistan over the course of four pandemic waves
Source: PLoS One. 2024 Nov 19;19(11):e0298940. doi: 10.1371/journal.pone.0298940 (PMC11575833; doi:10.1371/journal.pone.0298940)
Supplement: Attachment — Submitted filename: Response to Reviewers.docx [file pone.0298940.s004.docx]

**Response to Reviewers:**

**Response to Reviewer #1**

Dear reviewer,

We appreciate your thoughtful feedback on our article. Your comments were insightful, and we tried to cover them as much as we can. Below are our responses to the points you raised.

1. *In the introduction, it's essential to underscore that the emergence of new variants has facilitated the virus's evasion of detection using molecular assays (DOI: 10.1002/jmv.28241) and immune escape, leading to breakthrough infections post-vaccination (DOI: 10.1002/jmv.27688).*

Our response:

We appreciate your suggestion regarding the introduction section. After reviewing the articles you recommended, we have tried to incorporate additional information on lines 116-118 in the Manuscript file (136-138 in the Revised Manuscript with Track Changes file), taking into account the focus of our research.

2. *Kindly emphasize the novelty of the present study amidst the existing genomic characterization data for SARS-CoV-2 and its variants.*

Our response:

Based on our obtained results, we tried to highlight the novelty of our present study on lines 52-54 in the Manuscript file (59-52 in the Revised Manuscript with Track Changes file).

3. *In the discussion section, highlight that efforts to detect new variants extended beyond clinical surveillance to include wastewater-based genome monitoring. This method rapidly detects viruses up to the strain level, enabling tracking of variant transmission routes and evolutionary dynamics (DOI: 10.1016/j.ijheh.2023.114224).*

Our response:

We appreciate the reviewer for emphasizing the significance of wastewater-based genomic surveillance. After reviewing the suggested article, we considered that wastewater-based genome monitoring would be one possible method that helps to increase the pandemic monitoring in Uzbekistan and preferred to highlight it in our manuscript on lines 463-467 in the Manuscript file (510-512 in the Revised Manuscript with Track Changes file).

4. *Discuss the necessity of characterizing emerging variants and their potential impact on long COVID development, now recognized as the most dreaded sequelae of acute SARS-CoV-2 infection. Reference studies such as DOI: 10.1016/j.jinf.2023.12.004 and DOI: 10.3390/v14122629.*

Our response:

After reviewing the articles you suggested, we have tried to give a brief information about long-COVID symptoms on 457-462 lines in the Manuscript file (502-507 in the Revised Manuscript with Track Changes file).

*5. Kindly address the limitations of the present study and provide recommendations to mitigate these limitations.*

Our response:

Thank you so much for your feedback, as we found it very reasonable. In the conclusion section (478-588 lines in the Manuscript file, 523-533 in the Revised Manuscript with Track Changes file), we have made an effort to address the limitations of our research and provide our insights on them.

Please, check the revision and suggest your opinion.

**Response to Reviewer #2**

Dear Mohamad S. Hakim,

We want to express our gratitude for the insightful feedback you provided on our article. Your comments were not only reasonable but also so valuable that we have made an effort to integrate them into our work. Please find below our responses to the comments you raised.

*Table 1: it is not known the type, total number, and the frequency of mutations detected in a specific VOC. The authors need to show the frequency of mutations within a specific VOC. As a reference, please see figure 3 of this publication: <https://pubmed.ncbi.nlm.nih.gov/38244104/> Those should be the main table/ figure, and for the original table 1, you can move it as supplementary table because the information is not that informative (it combines all sequence data from the four waves).*

Our response:

Thank you very much for your feedback on Table 1. After reviewing the suggested article, we found it helpful in creating a clear visualization to display the frequency of mutations found in our sequenced Delta and Omicron variants. Following your advice, we attempted to detail the mutations identified in a particular VOC in Table 1. As a result, Table 1 was reorganized, however, we would like to keep it in the manuscript 302-308 and 325-327 in (332-338 and 355-364 in the Revised Manuscript with Track Changes file).

Please, check this version and suggest your opinion.

*Table 2: it is also similar to Table 1. The current presentation is not informative. It is not known which mutation that is highly frequent (>10%) and which mutation that is rarely found. You can move it as a supplementary table. It is not known which mutation that belonged to a specific VOV (for example, mutation that is exclusively found in Delta but not in Omicron variant).*

Our response:

As you suggested we have rearranged Table 2 and move it to supplementary material.

*Line 96-98: The statement is not correct. The emergence of new strains is not associated with increased transmission and virulence of the new strains compared to previous strains.*

Our response:

After carefully read the statement, we realized that it is not applicable to all variants of SARS-CoV-2 and therefore inaccurate. Thus, we prefer to remove it.

*Line 277-280: Is that because the majority of samples sequenced were from the fourth wave? So, it is due to sampling bias. The authors should provide the details how many samples out of 110 samples belonged to a specific wave.*

Our response:

Thank you so much for your comment as we found it very reasonable. We tried to get the same number of samples from 2021 and 2022, however, out of 144 samples, we obtained 110 high-quality sequencing results (20A-3, Alpha-4, Delta-42, and Omicron-61) for analysis. As we could not take, the same number of samples from each wave, we acknowledge that we cannot assert that "the most common strains circulating in Uzbekistan belong to the Omicron variant...". Therefore, we have included information about the distribution of samples from each period (wave) on lines 242-244 in the Manuscript file (266-268 in the Revised Manuscript with Track Changes file).

We have reviewed and addressed the comments on the lines you have mentioned: Line 44, Line 232-233, Line 238, 241, Line 352, Line 356, Line 360, Line 378-380, Line 425, Line 56-59, 451-452, and the final ones. We have made efforts to correct them and incorporate the necessary revisions.

**Once again, we express our sincere gratitude to the reviewers.**

Kind regards,

Esonova Gulnoza

PhD student and junior researcher

Biotechnology Laboratory of Center for Advanced Technologies.
 [esonovagulnoza96@gmail.com](mailto:esonovagulnoza96@gmail.com)
