# Supplementary material for: Complete genome sequencing of SARS-CoV-2 strains that were circulating in Uzbekistan over the course of four pandemic waves
Source: PLoS One. 2024 Nov 19;19(11):e0298940. doi: 10.1371/journal.pone.0298940 (PMC11575833; doi:10.1371/journal.pone.0298940)
Supplement: Attachment — Submitted filename: Response to Reviewers.docx [file pone.0298940.s005.docx]

**Response to Reviewers:**

**Response to Reviewer #3**

Dear reviewer,

We are grateful for your insightful feedback on our article. Your comments were not only reasonable but also so valuable that prompting us to incorporate them into our work. We have made every effort to address all your comments. Below are our responses to the points you raised.

*Your major concerns:*

1. *L221, “Standard bioinformatic tools” is not quite specific enough. Obviously DRAGEN can analyze the samples prepared with the COVIDSeq workflow, however, the DRAGEN pipeline would not be appropriate to analyze the results from the CleanPlex (Paragon) workflow…*

Thank you so much for your comment. After reviewing your concern, we tried to clarify and specify the bioinformatic part (Lines 222-233 in the Manuscript file).

2. *The second major concern is the discussion of limitations.*

We appreciate your comment about the limitations, but since we are currently working on another article that examines the advantages and disadvantages of two high-throughput SARS-CoV-2 sequencing methods (CleanPlex and COVIDSeq) along with all the research limitations, we preferred not to stop too much on them. Therefore, in order not to deviate from the main topic of this article, we preferred to briefly address the limitations and not re-label the "Conclusion" section.

In our previous study, we provided detailed information about ORF7a: P45L and its association with disease severity [16]. In this article, we hypothesized that the absence of the ORF7a: P45L mutation in Omicron variants could be a reason for the decrease in virulence in Omicron variants (Lines 131-135 in the Manuscript file).

3. *About the ref 44.*

We thoroughly reviewed this section of our article - how wastewater could be useful in surveillance of viruses. After carefully reviewed, we agreed with your opinion and, to prevent any further misunderstandings, have decided to remove ref 44.

Here, our responses to your minor concerns:

1. *L106, why is May 2021 not considered a “peak”?*

The peaks were primarily determined by the number of cases, and May 2021 was not considered a peak as the number of cases fell below 500 (also low death rate) unlike the other peaks. In addition, the state did not show great concern at that time (May 2021) and the sending of samples to the center did not increase.

2. *L124, this is a stylistic suggestion, but I believe the introduction could end here (with “…preventive measures in Uzbekistan.”) The rest of the paragraph lists specific findings that are included in the results; and, in particular, the last sentence is discussion/conclusion without direct evidence presented in this study.*

We appreciate your suggestion, but we have preferred to keep this part unchanged.

3. *L132, P45L is ambiguous – which gene/orf? (ORF7a?) Check to make sure that all substitutions/indels are labeled as "gene:A##B", or that their reference gene is otherwise unambiguous.*

We clarified that the P45L substitution belongs to the ORF7a gene. (Line 132 in the Manuscript file)

4. *L176-177, I hope the authors realize that the “RT” in “RT-PCR” stands for “reverse transcription” and the “q” in “RT-qPCR” indicates it is a “real-time” assay, even if the result is semi-quantitative. Therefore, in this case, “RT-qPCR” is “real-time reverse transcription PCR”. See the MIQE guidelines. It seems clear in L188-189, but here it is a bit conflated.*

We noted and revised this abbreviation terminology (Lines 176 and 179 in the Manuscript file).

5. *L183 & L186, what is the difference between “CleanMag®” and “CleanPlex”?*

Thank you for your careful review of our article. We made a technical error: CleanMag® refers to the magnetic beads used for purification, while CleanPlex is the complete kit. We have corrected this in line 183 (manuscript file).

6. *L302 (“The increase in the number…”), L317 (“This shows that…”) and elsewhere: these are discussion points.*

We appreciate your suggestion and will try to consider and apply it in our future work.

7. *Table 1 is now two tables, somehow - the authors should fix the formatting. Note that the some rows of the “Spike” portion of Table 1 contain duplicated sequence features.*

We identified the duplicates and removed them.

8. *L332 and elsewhere – is this nsp12: P314L? nsp12:K323 has been largely unchanged. I think the authors should re-evaluate their conclusions about this mutation. The website they cite (ref. 14, not a peer-reviewed scientific source) does not mention this mutation. However, there have been several peer-reviewed scientific publications that discuss this specific mutation.*

No, it is not nsp12: P314L. In line 332, we discussed the P323L substitution in the NSP12 protein (this mutation is also shown in S1 Table). In our results, we even did not identify such substituns : P314L and K323. Additionally, we did not reference ref. 14 for this mutation, as you mentioned. For the NSP12: P323L substitution, we cited ref. 21.

9. *L358, where is the increased “rate” of infection shown?*

In Figure 1.

10. *L437, I am confused by this sentence and the following paragraph. nsp13:R392C was “reported for the first time in this study”, but was previously studied [ref 41]? Actually, ref 41 (Kuman et al., published since March 2023 in Vacunas, please update the reference) does discuss nsp13, but does not identify this mutation. However, the authors seem to be referring to Hossain et al. 2022 (Microb Pathog) [ref 34], which discusses this mutation in nsp13 in their review of “all” nonstructural mutations associated with Omicron (although they are not clear about how they identified the mutations),*

To clarify any confusion, we rearranged this sentence. We also updated reference 41. While we intended to refer to reference 22 about the mutation, we mistakenly cited reference 41. We have also revised and corrected this error. Thank you for your feedback.

Regarding the reference, we have revised and reorganized it based on your suggestion. We have removed ref 8 and ref 44. We have also checked the references whether are up to date and updated ref 35 and 40.

**Thank you so much for carefully reviewing our article. We tried to cover all your comments and other suggestions (about the references and editing) on the manuscript.**

**Please, check the revision and suggest your opinion.**

**Response to Reviewer #4**

Dear Burtram Clinton Fielding,

We are pleased with your comments on our article. We found them reasonable and took them into consideration. Below, you can find our responses to the points you raised.

1. *The authors must be commended for addressing the comments raised by the previous reviewers.*

We appreciate your acknowledgment of our work.

2. *This is an important article showing the genomic mutations of SARS-COV-2 strains circulating in Uzbekistan. However, the title does not (really) convey the content and findings. Should the title not read, "**Complete genome sequencing of SARS-COV-2 strains that were circulating in Uzbekistan during the third and fourth pandemic waves in 2021 and 2022"? The authors state, "We tried to get the same number of samples from 2021 and 2022", and later refer to waves 3 and 4. This supports my recommendation to change the title.*

We have taken your offer into account; however, as we aimed to summarize the research on whole genome sequencing of SARS-CoV-2 in Uzbekistan, we encountered previous results that we used for comparison with our latest data. Therefore, we preferred to not change the title.

3. *The previous reviewers commented: "Line 96-98: The statement is not correct. The emergence of new strains is not associated with increased transmission and virulence of the new strains compared to previous strains." The authors responded, "After carefully read the statement, we realized that it is not applicable to all variants of SARS-CoV-2 and therefore inaccurate [sic]. Thus, we prefer to remove it." However, I believe that this is an important concept and I advise the authors not to remove the statement, but rather to amend it, eg "The emergence of new strains is more often associated with increased transmission, but not necessarily incfreased virulence of the new strains compared to previous strains"*

We greatly appreciate your advice and find it reasonable after reviewing the reference you provided. However, since the idea you mentioned could reach to the readers while reading the article, we preferred not to make any changes to this part.

**Once again, we express our sincere gratitude to the reviewers.**

Kind regards,

Esonova Gulnoza

PhD student and junior researcher

Biotechnology Laboratory of Center for Advanced Technologies.
 [esonovagulnoza96@gmail.com](mailto:esonovagulnoza96@gmail.com)
